# Anti-Cancer Activity of Lobaric Acid and Lobarstin Extracted from the Antarctic Lichen *Stereocaulon alpnum*

**DOI:** 10.3390/molecules23030658

**Published:** 2018-03-14

**Authors:** Ju-Mi Hong, Sung-Suk Suh, Tai Kyoung Kim, Jung Eun Kim, Se Jong Han, Ui Joung Youn, Joung Han Yim, Il-Chan Kim

**Affiliations:** 1 Division of Polar Life Sciences, Korea Polar Research Institute, Incheon 21990, Korea; wnal5555@kopri.re.kr (J.-M.H.); tkkim@kopri.re.kr (T.K.K.); je2202@kopri.re.kr (J.E.K.); hansj@kopri.re.kr (S.J.H.); ujyoun@kopri.re.kr (U.J.Y.); jhyim@kopri.re.kr (J.H.Y.); 2 Department of Biosciences, Mokpo National University, Muan 58554, Korea; sung-suk.suh@kopri.re.kr; 3 Department of Pharmacy, Graduate School, Sungkyunkwan University, Suwon 16419, Korea; 4 Department of Polar Sciences, University of Science and Technology, Incheon 21990, Korea

**Keywords:** lobaric acid, lobarstin, antarctic lichen, apoptosis, cell cycle arrest, human cervix adenocarcinoma, human colon carcinoma

## Abstract

Lobaric acid and lobarstin, secondary metabolites derived from the antarctic lichen *Stereocaulon alpnum*, exert various biological activities, including antitumor, anti-proliferation, anti-inflammation, and antioxidant activities. However, the underlying mechanisms of these effects have not yet been elucidated in human cervix adenocarcinoma and human colon carcinoma. In the present study, we evaluated the anticancer effects of lobaric acid and lobarstin on human cervix adenocarcinoma HeLa cells and colon carcinoma HCT116 cells. We show that the proliferation of Hela and HCT116 cells treated with lobaric acid and lobarstin significantly decreased in a dose- and time-dependent manner. Using flow cytometry analysis, we observed that the treatment with these compounds resulted in significant apoptosis in both cell lines, following cell cycle perturbation and arrest in G2/M phase. Furthermore, using immunoblot analysis, we investigated the expression of cell cycle and apoptosis-related marker genes and found a significant downregulation of the apoptosis regulator B-cell lymphoma 2 (Bcl-2) and upregulation of the cleaved form of the poly (ADP-ribose) polymerase (PARP), a DNA repair and apoptosis regulator. These results suggest that lobaric acid and lobarstin could significantly inhibit cell proliferation through cell cycle arrest and induction of apoptosis via the mitochondrial apoptotic pathway in cervix adenocarcinoma and colon carcinoma cells. Taken together, our data suggests that lobaric acid and lobarstin might be novel agents for clinical treatment of cervix adenocarcinoma and colon carcinoma.

## 1. Introduction

Cancer is one of the most common diseases around the world [1]. According to the World Health Organization, cancer is the second leading cause of human death globally and this disease had an incident rate of more than 14.1 million new cases and 8.2 million deaths in 2012 [2]. In recent decades, many scientists around the world have devoted their efforts to cancer study, and various effective therapies have been developed to treat cancer. However, there are several issues in cancer treatment that need to be solved. For example, chemotherapy can kill cancer cells, but it can also affect normal cells that grow or divide rapidly, such as new blood cells in the bone marrow and cells in the mouth, stomach, skin, hair, and reproductive organs. As normal cells become damaged because of chemotherapy, side effects such as diarrhea, nausea, and memory loss can occur [3,4]. Therefore, it is crucial to establish valid therapeutic regimes that are both safe and effective, and identify anticancer agents with few side effects, low toxicity, and high anticancer potency [5,6].

Lichens are symbiotic associations between fungi and algae. A number of lichens have been used for various purposes in folk medicine by many cultures across the world, particularly in temperate and arctic regions [7,8]. Lichen metabolites have many biological properties such as antibiotic, antitumor, anti-inflammatory, antiviral, antifungal, enzyme inhibitory, and plant growth inhibitory properties, suggesting that they can be a potential source of chemicals in the pharmaceutical industry or agriculture [9,10]. For example, it has been reported that the adjuvant treatment with lichen-derived usinic acid and zinc sulphate after radiation therapy accelerates reepithelization and reduces recurrence in cervical cancer caused by human papillomavirus (HPV) [11]. In addition, some studies indicate that usinic acid is a new non-toxic anticancer drug because it reduces the proliferation of human breast cancer cells and lung cancer cells without DNA damage [12].

**Figure 1:**
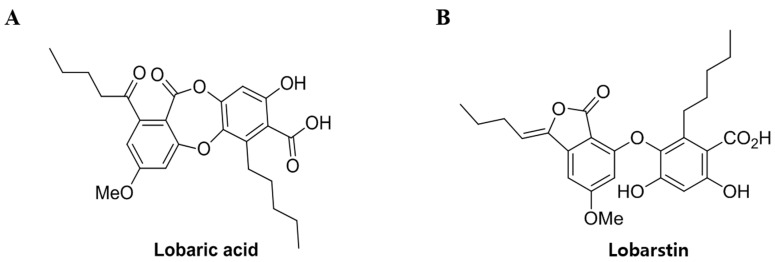
Chemical structure of (**A**) lobaric acid (molecular weight [Mw], 456.49) and (**B**) lobarstin (Mw. 456.49).

Lobaric acid and lobarstin, secondary metabolites isolated from the Antarctic lichen *Stereocaulon alpnum*, are biologically potent bioactive compounds with antibiotic, antimycobacterial, antiviral, and antipyretic properties (Figure 1) [13,14]. In addition, it has been demonstrated that they have various biological activities such as anti-proliferative, anti-inflammatory, antioxidant, antimicrobial effect and a therapeutic effect on glioblastoma [15,16,17]. For example, lobarstin are cytotoxic in human glioblastoma T98T cells treated with low concentrations of these compounds (below 100 μM) for 48 h [18]. However, the effect of lobaric acid and lobarstin on anticancer activity against other cancer cell lines, including cervix adenocarcinoma cells (HeLa) and colon carcinoma cells (HCT116), has not yet been investigated and characterized.

The purpose of this study was to investigate the anticancer effect of lobaric acid and lobarstin isolated from Antarctic lichens in HeLa and HCT116 cells. In particular, we first examined the molecular mechanism underlying the cell cycle arrest and apoptosis induced by lobaric acid and lobarstin. Our data suggests that these compounds might be effective anticancer agents. In HeLa and HCT116 cells, lobaric acid and lobarstin seem to be more effective than the known anticancer drug doxorubicin (DOX), showing a significant inhibition of cell proliferation via modulation of the expression of crucial regulators of cell cycle and apoptosis.

## 2. Results

### 2.1. Effects of Lobaric Acid and Lobarstin on Cell Viability

**Figure 2:**
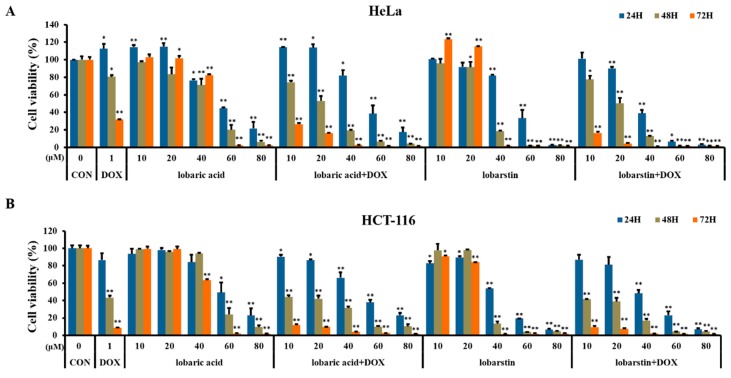
Effect of lobaric acid and lobarstin on the growth of (**A**) HeLa and (**B**) HCT116 cells. The cells were treated with the indicated concentrations of lobaric acid and lobarstin for 24, 48, or 72 h. Cell growth was measured with a cell proliferation assay. Data are presented as percentage of control and are the mean ± SEM (*n* = 3); * *p* < 0.05 and ** *p* < 0.01 compared to the control.

To investigate the cytotoxic effect of the lichen-derived metabolites lobaric acid and lobarstin, we evaluated the viability of HeLa and HCT116 cells exposed to these compounds using the MTT method. Both HeLa and HCT116 cells were treated with various concentrations of lobaric acid or lobarstin (10, 20, 40, 60, and 80 μM) for 24, 48, and 72 h, or with DOX (1 μM, used as positive control). The viability of the cells in response to the treatment with lobaric acid and lobarstin was significantly decreased in a dose-dependent and time-dependent manner when compared with untreated control (Figure 2), with a half maximal inhibitory concentration (IC_50_) of 50 μM in both cell lines. Next, to investigate the possible synergistic effect of these compounds with DOX, both cell lines were simultaneously treated with DOX and lobaric acid or lobarstin. The proliferation of Hela and HCT116 cells treated with DOX and one of the lichen-derived metabolites investigated decreased more significantly than that of the cells treated with lobaric acid or lobarstin alone. These data suggest that lobaric acid and lobarstin have cellular toxicity on cancer cells and suppressed their growth synergistically with DOX. These data suggest that lobaric acid and lobarstin have cellular toxicity on cancer cells and suppressed their growth synergistically with DOX.

### 2.2. Effect of Lobaric Acid and Lobarstin on Cell Morphology

**Figure 3:**
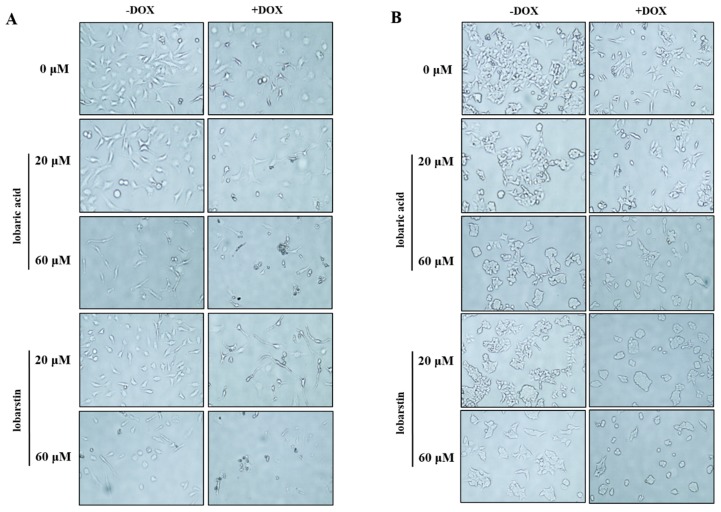
Morphological changes of (**A**) HeLa and (**B**) HCT116 cells treated by lobaric acid and lobarstin at the concentration of 20 and 60 μM for 24 h. Light microscopy photographs are shown (20× objective).

To better understand the effect of lobaric acid and lobarstin on HeLa and HCT116 cells, their morphology before and after the treatment with these compounds was observed using an optical microscope (Figure 3). The untreated cells were uniformly distributed on a cultured field, exhibiting a polygonal shape. After treatment of HeLa cells with different concentrations (20 and 60 μM) of lobaric acid and lobarstin for 24 h, the shape of these cells dramatically changed to an elongated shape with filamentous protrusions: the magnitude of the change observed depended of the metabolite concentration used. Additionally, treatment of HCT116 cells with lobaric acid and lobastin transformed the cells from polygonal to circular. This morphological change is one of the typical signs of apoptosis, and it is usually accompanied by cell shrinkage, fragmentation into membrane-bound apoptotic bodies, and rapid phagocytosis by adjacent cells [19]. Furthermore, a significant reduction in cell number and the increase in the number of floating cells was observed in both HeLa and HCT116 cells. Furthermore, consistent with the synergistic effect of DOX and lobaric acid or lobarstin, the concomitant treatment with DOX (1 μM) and one of the lichen-derived metabolites was associated with a bigger reduction in cell number and more pronounced morphological changes than the treatment with lobaric acid or lobarstin.

### 2.3. Effect of Lobaric Acid and Lobarstin on Apoptosis

**Figure 4:**
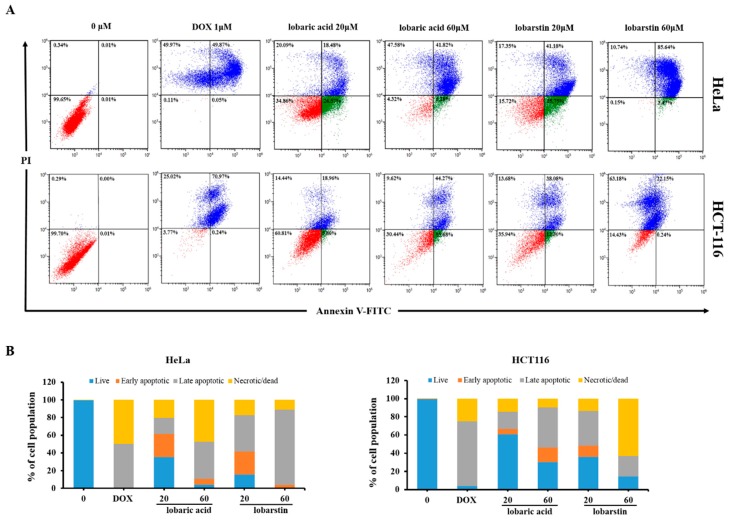
Effect of lobaric acid and lobarstin on the apoptosis of HeLa and HCT116 cells. The cells were stained with annexin V/propidium iodide (PI), and the apoptotic cell population was evaluated by flow cytometry (**A**). The graphical representation of the percentage of live, early apoptotic, late apoptotic, and necrotic/dead cells is shown (**B**).

**Figure 5:**
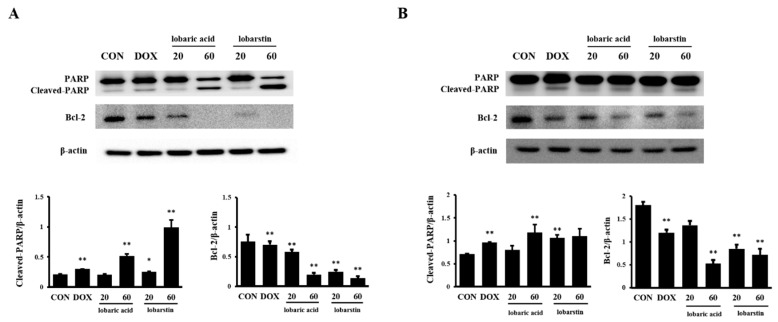
Effect of lobaric acid and lobarstin on the protein levels of cleaved PARP and Bcl-2 in HeLa and HCT116 cells. (**A**) HeLa and (**B**) HCT116 cells were treated with different concentrations of lobaric acid and lobarstin (20 or 60 μM) for 24 h. The expression of the indicated proteins was investigated by western blot analysis; β-actin was used as loading control. The densitometry value of each band was determined with the Image J software. Data are presented as the mean ± SEM of duplicate independent experiments; * *p* < 0.05 compared with control; ** *p* < 0.01 compared with control.

Next, we verified whether the cytotoxic effect of lobaric acid and lobarstin on cancer cells occurred via apoptosis. For this purpose, we performed flow cytometric analysis. As shown in Figure 4, the population of apoptotic cells significantly and dose-dependently increased after treatment with lobaric acid or lobarstin: in HeLa cells the percentage of Annexin V-positive cells increased to 45.0% and 48.1% after treatment with 20 μM lobaric acid and lobarstin, respectively; and to 66.9% and 89.11% after treatment with 60 μM of the same compounds, respectively; in HCT116 cells, similar treatments were associated with the following percentages of Annexin V-positive cells: and 24.7%, 59.9%, 50.3% and 22.4% (cells treated with lower or higher concentrations of lobaric acid and lobarstin). The Annexin V/PI staining allows the discrimination between early and late apoptotic cells (Annexin V-positive and Annexin V- and PI-positive, respectively). We found that early apoptosis occurs at a lower concentration (20 μM) of lobaric acid and lobarstin, and late apoptosis occurs at a higher concentration (60 μM) (Figure 4). Next, to further understand the mechanisms of lobaric acid- and lobastin-induced apoptosis, we investigated the expression of major regulators of apoptosis upon treatment of the cells with these compounds. Consistent with the flow cytometry analysis, we found that lobaric acid or lobarstin dose-dependently increased PARP cleavage and decreased the expression of Bcl-2 (Figure 5), both of which play an important role in promoting cell survival and inhibiting the action of pro-apoptotic proteins. Taken together, these data suggest that lobaric acid and lobarstin significantly induce apoptosis in HeLa and HCT116 cells in dose-dependent manner.

### 2.4. Effect of Lobaric Acid and Lobarstin on the Cell Cycle

To elucidate whether the growth inhibitory effect of lobaric acid and lobarstin on HCT116 cells was partly due to cell cycle arrest, we performed cell cycle analysis using PI staining.

**Figure 6:**
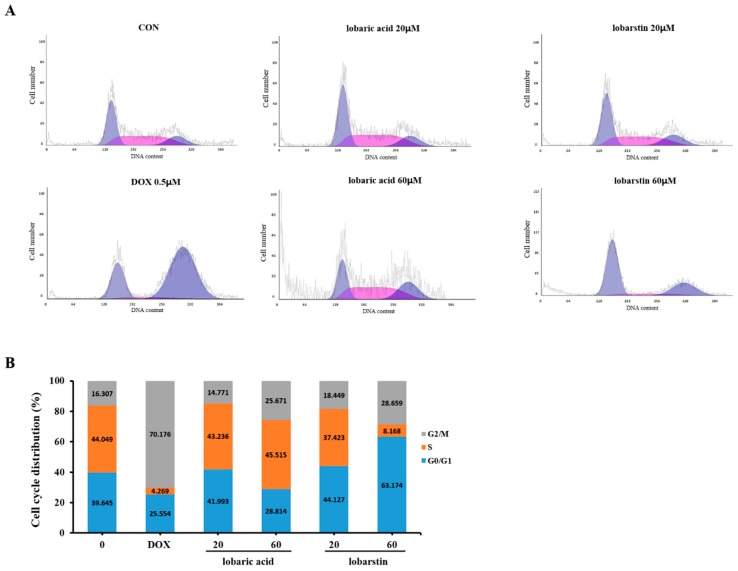
Effect of lobaric acid and lobarstin on HCT-116 cell cycle distribution. (**A**) HCT116 cells were treated with lobaric acid and lobarstin for 24 h, stained with propidium iodide (PI), and analyzed using a flow cytometer; (**B**) the histogram indicates the percentages of total cells in each phase of the cell cycle.

HCT116 cells were treated with two different concentration of lobaric acid and lobarstin, 20 and 60 μM, for 24h. DOX (0.5 μM) was used as a positive control. As shown in Figure 6, the percentage of cells in G2/M phase increased dose-dependently, showing 16.3%, 14.7% and 25.67% of G2/M population at 0, 20, and 60 μM lobaric acid, respectively. Similar results were obtained upon treatment with lobarstin (16.3%, 18.4% and 28.6% of cells in G2/M at 0, 20, and 60 μM, respectively) Taken together, these results suggest that lobaric acid and lobarstin increase the proportion of cells in G2/M phase, leading to the inhibition of cellular proliferation.

## 3. Discussion

Several tumor cell lines acquire molecular mechanisms to suppress apoptosis and resist the treatment with apoptotic agents [20]. Apoptosis, also called programmed cell death, plays an important part in many biological events, including morphogenesis, cell turnover, and removal of harmful cells. Therefore, it is not surprising that this process is tightly controlled and defects in apoptosis can cause cancer, autoimmune diseases, or degenerative diseases [21,22]. The cell cycle—in which cyclin-dependent kinases (CDKs) play a key role in controlling its initiation, progression, and completion—is frequently dysregulated in human cancers. The relationship between cyclins and CDK inhibitors, phosphorylation status of the CDKs, and their ubiquitin-mediated proteolysis, modulate the activity of these proteins and allow the orderly transition between cell cycle stages. As malignant cells evolve, genetic and post-transcriptional mechanisms generally affect the expression of cell cycle regulatory proteins, leading to the overexpression of cyclins and the loss of CDK inhibitors, and consequent cellular damage. Cell cycle arrest frequently occurs at G1/S or G2/M transitions [23].

In the present study, to explore the mechanism responsible for the anti-proliferative effect of lobaric acid and lobarstin, we examined the apoptosis and cell cycle of human cervix adenocarcinoma (HeLa) and human colon carcinoma cells (HCT116), which harbor wild type *TP53* gene. According to our data, despite their structural differences (Figure 1), lobaric acid and lobarstin induced similar degrees of apoptosis and arrest in the G2 phase of the cell cycle in a dose dependent manner, suggesting that the structural differences between them do not affect their anticancer activity.

In general, the apoptotic pathway consists of multiple sequential molecular events including the modulation of apoptosis-related genes. Members of the Bcl-2 family are key regulatory factors in apoptosis. It has been reported that the Bcl-2 family regulates mitochondrial membrane permeability and apoptosis [24,25]. In addition, PARP is a stress response protein that repairs damaged DNA and regulates chromatin structure by poly ADP-ribosylating nuclear proteins. Proteolytic cleavage of PARP by caspases is an early indicator of apoptosis [26]. We found that lobaric acid and lobarstin induced apoptosis by significantly downregulating Bcl-2 and upregulating cleaved PARP. Furthermore, previous studies showed that treatment with lobaric acid (20 μg/mL, exposure time longer than 48 h) effectively inhibits the growth of breast cancer T-47D and ZR-75-1 cells, with an IC_50_ of 25 and 46 μg/mL, respectively [15]. A different study showed that exposure to 25 μM lobaric acid for 48 h exerts an inhibitory effect on the proliferation of HeLa and HCT116 cells; the IC_50_ were 78.0 ± 7.1 μM and 93.2 ± 0.2 μM, respectively [27]. It has been reported that lobarstin IC_50_ is approximately 60 μM in human glioblastoma T98G cells treated with this compound for 72 h. In our study, treatment with 20 μM lobaric acid for 48 h did not significantly induce apoptosis in Hela and HCT116 cells (Figure 2). In addition, in the high concentration treatment of lobarstic acid (60 μM), apoptotic cell population of HCT116 was higher than that observed in HeLa cells, whereas population of apoptotic cells was lower at low concentration (20 μM). On the other hand, apoptosis was severely induced in lobarstin-treated HeLa cells compared with HCT116, and a significant increase of necrotic cells was observed in HCT116. These diverse effects on apoptosis might be caused by difference cellular contexts of HeLa and HCT116. In addition, the data from apoptosis analysis was supported by the morphological observations of the lobaric acid- or lobarstin-treated cancer cells was supported by the apoptosis analysis, showing that visible changes in cell morphology indicative of cell death such as shrinking at the dose dependent manner.

To test the effect of lobaric acid and lobarstin on the proliferation of HCT116 cells, we performed cell cycle analyses. Our results showed that treatment with 60 μM of lobaric acid or lobarstin for 24 h induced a G2/M arrest in HCT116 cells compared to the control group. The proportion of cells in G2/M phase increased from 16.30% to 25.67% and 28.66% in the cells treated with lobaric acid and lobarstin, respectively. However, no changes in the cell cycle were observed in HCT116 cell treated with 20 μM lobaric acid or lobarstin, compared with control group. These results demonstrate that lobaric acid and lobarstin increase the proportion of cells arrested in G2/M in a dose-dependent manner. In the cell cycle, the G2/M checkpoint is a critical control point to attempt repairing DNA damage before cell division begins. Our data suggest that lobaric acid and lobarstin induce apoptosis through the arrest of human colon cancer cells in G2.

In conclusion, our data indicate that lobaric acid and lobarstin dose- and time-dependently inhibit cell proliferation and induce apoptosis in HeLa and HCT116 cells through cell cycle arrest at the G2/M phase transition and the modulation of apoptosis-related marker genes such as Bcl-2 and PARP. Taken together, our data suggest that lobaric acid and lobarstin are novel anticancer drugs for the treatment of human cervix adenocarcinoma and human colon carcinoma.

## 4. Materials and Methods

### 4.1. Preparation of Compounds

*Stereocaulon alpinum* was obtained from the Korea Polar Research Institute (KOPRI) (Incheon, Korea). For in vitro studies, lobaric acid and lobarstin were dissolved in dimethyl sulfoxide (DMSO, 40 mM stock stored at −20 °C) and diluted at the concentrations indicated. DOX was purchased from Sigma-Aldrich (St. Louis, MO, USA) and dissolved in DMSO.

### 4.2. Cell Culture

HeLa and HCT-116 cells were maintained in Dulbecco’s Modified Eagle medium (DMEM, Sigma-Aldrichm, St. Louis, MO, USA) supplemented with 10% heat-inactivated fetal bovine serum (FBS, Invitrogen, Burlington, ON, Canada), 1% (*w*/*v*) of an antibiotic-antimycotic solution (Invitrogen, Grand Island, NY, USA) in a 95% air and 5% CO_2_ humidified atmosphere at 37 °C.

### 4.3. Cytotoxicity Assay

HeLa and HCT116 cells were seeded at a density of 1 × 10^5^ cells/mL in 96-well plates, and incubated at the presence of various concentrations of lobaric acid and lobarstin for 24 h. Cell viability was determined by assessing the mitochondrion-dependent reduction of 3-(4,5-Dimethyl-2-thiazolyl)-2,5-diphenyl-2*H*-tetrazolium bromide (MTT, Amresco, Solon, OH, USA) to formazan. Briefly, 5 μL of a 5 mg/mL MTT solution were added to the cells, for 4 h at 37 °C. DMSO was added after removal of the medium. The optical density of formazan solution was then measured using a microplate reader (Thermo Scientific Inc., San Diego, CA, USA) at 570 nm. The levels of formazan generated by untreated cells were set as 100%.

### 4.4. Morphological Analysis

To investigate potential morphological changes, HeLa and HCT116 cells were seeded at a density of 2.5 × 10^5^ cells/mL in 12-well plates and incubated for 24 h before treatment. After 24 h, cells were treated with the indicated concentrations of lobaric acid or lobarstin and treated with 1 μM doxorubicin served as positive control for apoptotic morphology. The morphological changes in HeLa and HCT116 cells were observed under an inverted phase contrast microscope (EVOS, Seattle, WA, USA) after 24 h of treatment.

### 4.5. Apoptosis Assays

Apoptosis and/or necrosis were investigated in HeLa and HCT116 cells using Annexin V/fluorescein isothiocyanate (FITC) and propidium iodide (PI) double staining. Briefly, 2.5 × 10^5^ cells/well were seeded in 6-well plates. After 24 h, cells were treated with lobaric acid and lobarstin at the indicated concentrations for 24 h. The cells were then washed, harvested, and stained with Annexin V/FITC and PI (BD Biosciences, San Jose, CA, USA) according to the manufacturer’s instructions. Untreated cells stained with PI or Annexin V/FITC served as control. The samples were analyzed using a flow cytometer (Beckman Coulter Inc., Brea, CA, USA).

### 4.6. Immunoblot Analysis

Lobaric acid-treated, lobarstin-treated, and untreated HeLa and HCT116 cells were scraped and lysed in 100 μL of lysis buffer containing protease inhibitors (Roche Diagnostics GmbH, Mannheim, Germany). Protein concentrations in supernatants were determined using the Bradford reagent (Bio-Rad Laboratories Inc., Woodinville, WA, USA). Total proteins (25 μg) were separated by sodium dodecyl sulfate polyacrylamide gel electrophoresis (SDS-PAGE) on 10% gel, and separated proteins were transferred to a polyvinylidene fluoride (PVDF) membrane (Millipore, Billerica, MA, USA). The membrane was incubated with a blocking solution (5% skim milk in TBST), followed by overnight incubation at 4 °C with an appropriate primary antibody. The following primary antibodies and dilutions were used: anti-poly (ADP-ribose) polymerase (PARP, 1:1000 dilution, Cell Signaling, Danvers, MA, USA), anti-B-cell lymphoma 2 (Bcl-2, 1:1000 dilution, Abfrontier, Seoul, Korea), and β-actin (1:1000 dilution, Cell Signaling, Danvers, MA, USA). Membranes were washed three times with a Tris-buffered saline containing 0.1% Tween 20 (TBST), and then incubated with a 1:2000 dilution of horseradish peroxidase (HRP)-conjugated secondary antibody (Santa Cruz Biotechnology, Dallas, TX, USA) for 1 h at 20–25 °C. Membranes were washed three times with TBST, and then developed using an enhanced chemiluminescence (ECL) kit (Thermo Fisher, Waltham, MA, USA). For quantitative analysis, densitometric band values were determined using a Chemi-Doc instrument (Bio-Rad, Hercules, CA, USA).

### 4.7. Cell Cycle Analysis

HCT-116 cells were seeded in six-well plates at a density of 2.5 × 10^5^ cells/mL, grown for 24 h, and treated with lobaric acid and lobarstin at the indicated concentrations. After 24 h of treatment, the cells were washed with phosphate-buffered saline (PBS), fixed with 70% ethanol and stored at −20 °C overnight. For analysis, cells were washed, resuspended in PBS, and incubated with 5 μL of RNase A (10 mg/mL) and 5 μL of PI (50 μg/mL) at 37 °C for 30 min. After incubation, they were analyzed using a flow cytometer (Beckman Coulter Inc., Brea, CA, USA) −20,000 ungated events were analyzed.

### 4.8. Statistical Analysis

Data are expressed as the mean ± the standard errors of the mean (SEM). Statistical difference was evaluated with the Student’s *t*-test. *P* values lower than 0.05 were considered to represent significant differences.
